# Endoscopic Findings in Patients Presenting with Upper Gastrointestinal Bleeding

**DOI:** 10.7759/cureus.4280

**Published:** 2019-03-19

**Authors:** Saleh Mohammad, Bashir Chandio, Abrar Shaikh, Aftab A Soomro, Amber Rizwan

**Affiliations:** 1 Internal Medicine, Ghulam Muhammad Meher Medical Hospital, Sukkur, PAK; 2 Pathology, Ghulam Muhammad Meher Medical Hospital, Sukkur, PAK; 3 Family Medicine, Civil Hospital, Karachi, PAK

**Keywords:** upper gastrointestinal bleeding, peptic ulcer disease, variceal bleeding, upper gastrointestinal endoscopy, emergency medicine, esophageal varices

## Abstract

Introduction: Upper gastrointestinal bleeding (UGIB) is one of the most common and grave emergencies encountered by the emergency medicine doctors. The aim of this study is to assess the endoscopic findings in patients presenting with acute UGIB.

Methods: This is a retrospective study which included all endoscopy records of the Department of Gastroenterology, Ghulam Mohammad Maher Hospital, Sukkur from 1^st^ January 2017 till 30^th^ June 2018.

Results: There were 100 males (49.3%) and 103 females (50.7%) who underwent endoscopy in the study duration. The mean ± standard deviation (SD) age of the participants was 41.03 ± 14.94 years. Esophageal varices were found in 65% cases. There were more men (68%) with varices than women (32%). Almost 10% patients were with gastric erosions, 9% had antral gastritis, 6.4% had pangastritis, and peptic ulcer disease was found in 5.8% cases.

Conclusion: Variceal bleeding is the most common endoscopic finding in the patients with UGIB. Other lesser common causes include erosions of the gastric and esophageal mucosa.

## Introduction

Gastrointestinal (GI) bleeding remains one of the most common medical emergencies that require immediate intervention, resuscitation, and hospital admission [1]. Mostly it presents, acutely, as hematemesis (40%-50%)-bleeding site proximal to ligament of treitz; it may also present, chronically, as melena (70%-80%)-bleeding site distal to ligament of treitz; or a less common presentation hematochezia (10%)-fresh lower GI bleeding (LGIB) [2].

One hundred cases of upper GI bleeding (UGIB) per one hundred thousand population, annually have been reported. UGIB is four times more frequent than LGIB. It is associated with high morbidity; and mortality rate of almost 6%-10% [3]. The mortality predictors include advancing age, hemodynamic instability (hypotension, tachycardia), and presence of co-morbidities [4]. UGIBs are caused by erosive esophagitis, Mallory Weiss tears, esophageal varices, peptic ulcer disease (PUD), and gastric/duodenal erosions. Lesser common causes include aorto-enteric fistula, hemobilia, angiodysplasia, uremia, and coagulation disorders [5].

Esophageal varices and PUD remain the two most common etiologies of UGIB with a varying occurrence across the world. In our part of the world, esophageal varices are as common as in 50% cases; however, PUD is the leading cause in the West [6]. Esophageal varices are dilatations of the submucosal veins due to portal hypertension, which is commonly a result of liver cirrhosis. Esophageal varices are a significant risk factor of UGIB and a major cause of cirrhosis-related morbidity and mortality [2].

For determining the etiology of UGIB as well as intervening therapeutically, endoscopy has remained the foremost modality [7]. It is a relatively safer procedure and is commonly used in gastrointestinal settings. The aim of this study is to assess the causes of UGIB in our population based on their endoscopic findings.

## Materials and methods

This is a retrospective study which included all endoscopy records of the Department of Gastroenterology, Ghulam Mohammad Maher Hospital, Sukkur from 1st January 2017 till 30th June 2018. A total of 1,122 records were extracted after approval from the institutional review committee. Out of these, 118 records were excluded due to incomplete data. Remaining 1004 records were scanned for patients with UGIB/hematemesis as their indication to endoscopy. Two hundred and three records with UGIB (20.2%) that underwent emergency/elective endoscopy were included. Sixty five of these patients also had melena. Their age, gender, indication, and endoscopic finding were included. Data were entered and analyzed using SPSS v. 21. Frequency was calculated for stratified variables such as age, gender, history of nonsteroidal anti-inflammatory drugs (NSAIDs) and alcohol intake, and endoscopic findings; mean and standard deviation (SD) were calculated for continuous variables such as age.

## Results

There were 100 males (49.3%) and 103 females (50.7%). The mean ± SD age of the sample was 41.03 ± 14.94 years with 15 years as the minimum age and 75 years as the maximum age. Most of the patients were of age 41-60 years (n = 86; 42.4%), followed by age group 26-40 years (n = 60; 29.6%), less than 25 years (n = 42; 20.7%), and the least common age group was more than 60 years (n = 15; 7.4%) (Table 1). As seen in Table 2, the most frequent endoscopic finding in our study was varices; six of the patients with varices (4.5%) also had evidence of portal hypertensive gastropathy, which is also due to portal hypertension. Almost 10% patients were with gastric erosions which are an evidence of gastro-esophageal reflux disease. Antral gastritis (9%), pangastritis (6.4%), and peptic ulcer disease (5.8%) were also seen. The least common finding was esophagitis.

**Table 1:** Patient characteristics. SD, standard deviation; NSAIDs, non-steroidal anti-inflammatory drugs.

Patient characteristics	Number (%)
Gender	
Male	100 (49.3)
Female	103 (50.7)
Age in years	
Mean ± SD	41.03 ± 14.94
Less than 25	42 (20.7)
26-40	60 (29.6)
41-60	86 (42.4)
More than 60	15 (7.4)
First episode of hematemesis	95 (46.7)
History of NSAIDs use	35 (17.2)
Known case of hepatitis B/C	100 (49.3)
History of alcohol intake	59 (29.0)

**Table 2:** Endoscopic findings of the patients with upper gastrointestinal bleeding (UGIB).

Endoscopic diagnosis	Number (%)
Varices	132 (65.0)
Gastric erosions	20 (9.9)
Antral gastritis	18 (8.9)
Pangastritis	13 (6.4)
Peptic ulcer disease	12 (5.8)
Esophagitis	8 (3.9)

There were more males with variceal bleeding. In almost half of them, variceal bleeding was the first presentation of hepatitis B/C and 18% of them had previous episodes of UGIB (Table 3).

**Table 3:** Characteristics of patients with esophageal varices. UGIB, upper gastrointestinal bleeding.

Patient characteristics	Number (%)
Gender	
Male	90 (68.1)
Female	42 (31.8)
Viral serology status	
Known case of hepatitis B	32 (24.2)
Known case of hepatitis C	59 (44.6)
First presentation of Hepatitis B/C	72 (54.5)
History of previous episodes of UGIB	24 (18.1)

## Discussion

Patients presenting with UGIB had a mean age of 41 years. The male to female ratio was 1:1.03. The most common etiology behind UGIB in our study was esophageal varices (65%) which are related to portal hypertension secondary to liver cirrhosis. Other common causes included gastric erosions and gastritis. There were only 12 (5.8%) known cases of peptic ulcer disease.

Similar to our study, other studies have also reported comparable results. In a larger study conducted in Islamabad, 44.4% patients with UGIB had underlying esophageal varices and peptic ulcer was responsible for 19.4% cases only [8]. In another local study, 53% cases with UGIB had varices and 20% had PUD [9]. There is a rising trend of frequency of variceal bleeding with time and more recent studies are reporting higher percentages (72%) [10]. The difference in pattern can be justified by high incidence of viral hepatitis in this region. Esophageal varices not only complicate hepatitis but are also the first presentation of hepatitis in many cases as indicated in this study. Although the statistics are similar in India [11], in Iran PUD still remains a more common cause of UGIB [12].

Although the literature has given evidence of esophageal varices being almost twice as common in men as in women [1], our study showed an almost equal incidence. This discrepancy may be due to small sample size.

In our study, erosive diseases of the mucosa were the second most common cause of UGIB. Gastric erosions were more common as compared to esophageal erosions. In another local study, with esophageal varices being the most common endoscopic finding in UGIB (64%), erosive diseases of the mucosa were second in line with 15% erosive gastritis and 10% PUD [13].

The outcomes of our study indicate that with the causal use of proton pump inhibitors [14], the numbers are decreasing as far as peptic ulcer disease is concerned. However, with the rising trend in incidence of hepatitis B and C [15], more variceal bleeds are being encountered in the emergency departments. This study encourages population-based screening of hepatitis as well as emphasizes the need to take aggressive measures in order to combat the rising incidence of hepatitis in our setup.

## Conclusions

Variceal bleeding is a common endoscopic finding in patients with UGIB. Viral serology must be done in patients with UGIB especially those with esophageal varices on endoscopy. UGIB may be the first presentation of viral hepatitis in some patients. Other lesser common causes include erosions of the gastric and esophageal mucosa.
